# Supplementary material for: Causal Relationship between Aging and Anorexia Nervosa: A White-Matter-Microstructure-Mediated Mendelian Randomization Analysis
Source: Biomedicines. 2024 Aug 16;12(8):1874. doi: 10.3390/biomedicines12081874 (PMC11351342; doi:10.3390/biomedicines12081874)
Supplement: Supplementary file 1 [file biomedicines-12-01874-s001.zip › Supplementary Figure/Figure S4/Singe SNP plot of MR analysis for TL to ubm-b-1890.pdf]

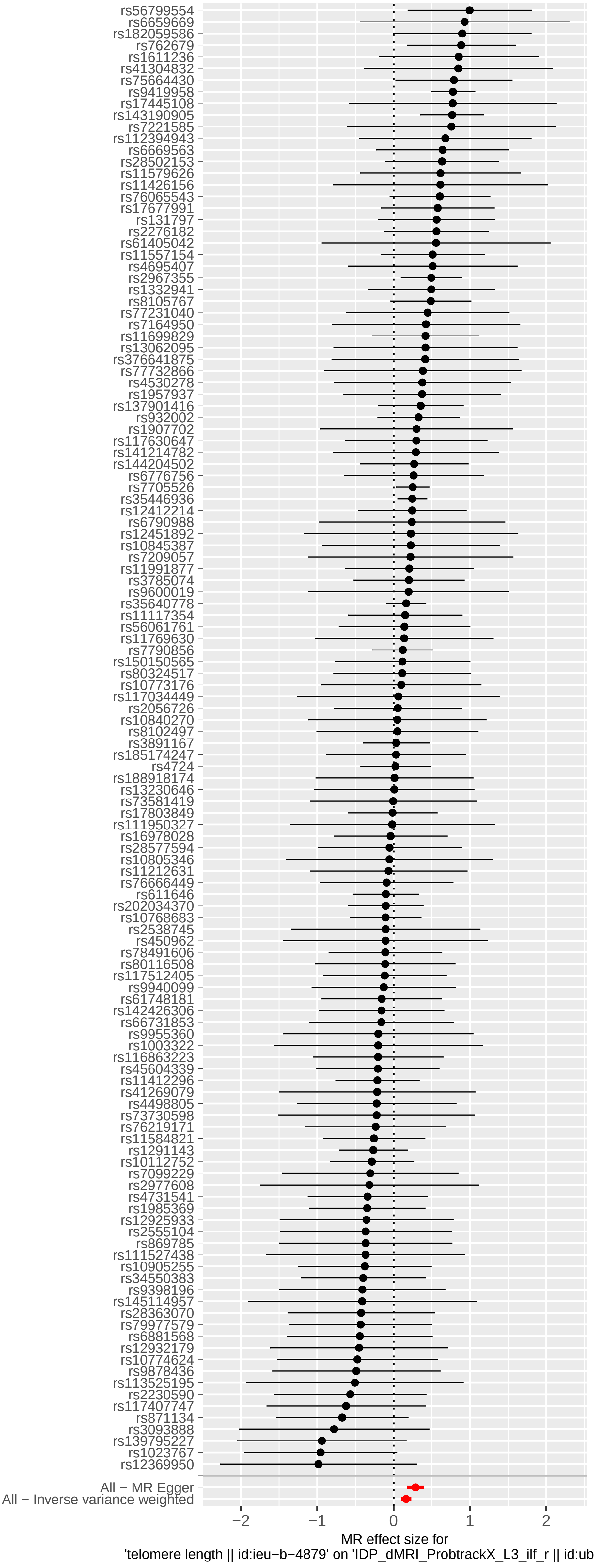

rs56799554
rs6659669
rs182059586
rs762679
rs1611236
rs41304832
rs75664430
rs9419958
rs17445108
rs143190905
rs7221585
rs112394943
rs6669563
rs28502153
rs11579626
rs11426156
rs76065543
rs17677991
rs131797
rs2276182
rs61405042
rs11557154
rs4695407
rs2967355
rs1332941
rs8105767
rs77231040
rs7164950
rs11699829
rs13062095
rs376641875
rs77732866
rs4530278
rs1957937
rs137901416
rs932002
rs1907702
rs117630647
rs141214782
rs144204502
rs6776756
rs7705526
rs35446936
rs12412214
rs6790988
rs12451892
rs10845387
rs7209057
rs11991877
rs3785074
rs9600019
rs35640778
rs11117354
rs56061761
rs11769630
rs7790856
rs150150565
rs80324517
rs10773176
rs117034449
rs2056726
rs10840270
rs8102497
rs3891167
rs185174247
rs4724
rs188918174
rs13230646
rs73581419
rs17803849
rs111950327
rs16978028
rs28577594
rs10805346
rs11212631
rs76666449
rs611646
rs202034370
rs10768683
rs2538745
rs450962
rs78491606
rs80116508
rs117512405
rs9940099
rs61748181
rs142426306
rs66731853
rs9955360
rs1003322
rs116863223
rs45604339
rs11412296
rs41269079
rs4498805
rs73730598
rs76219171
rs11584821
rs1291143
rs10112752
rs7099229
rs2977608
rs4731541
rs1985369
rs12925933
rs2555104
rs869785
rs111527438
rs10905255
rs34550383
rs9398196
rs145114957
rs28363070
rs79977579
rs6881568
rs12932179
rs10774624
rs9878436
rs113525195
rs2230590
rs117407747
rs871134
rs3093888
rs139795227
rs1023767
rs12369950
All − MR Egger
All − Inverse variance weighted
−2
−1
0
1
2
MR effect size for
'telomere length || id:ieu−b−4879' on 'IDP_dMRI_ProbtrackX_L3_ilf_r || id:ubm
